# Comparison of administration of oral versus Infusions intravenous iron therapy in diabetic patients with chronic kidney disease receiving Erythropoietin

**DOI:** 10.12669/pjms.41.8.12022

**Published:** 2025-08

**Authors:** Nizamud Din, Ayesha Durrani, Muhammad Kashif Nouman, Bushra Haider

**Affiliations:** 1 Nizamud Din Senior Registrar, Endocrinology Department, Northwest General Hospital and Research Centre, Northwest School of Medicine, Peshawar, Pakistan; 2 Ayesha Durrani Postgraduate Trainee, Endocrinology Department, Northwest General Hospital and Research Centre, Northwest School of Medicine, Peshawar, Pakistan; 3 Muhammad Kashif Nouman Medical Officer, Department of General Medicine, Northwest General Hospital and Research Centre, Northwest School of Medicine, Peshawar, Pakistan; 4 Bushra Haider Research Officer, Medical Professional Educational Department, Northwest General Hospital and Research Centre, Northwest School of Medicine, Peshawar, Pakistan

**Keywords:** Anemia, Diabetes Mellitus, Erythropoietin, Iron-Deficiency, Kidney Diseases

## Abstract

**Objective:**

To compare the efficacy of administration oral versus infusions intravenous iron (IV) therapy in diabetic patients with chronic kidney disease (CKD) receiving Erythropoietin.

**Methods:**

This retrospective comparative study, including 144 diabetic and CKD patients (72 in each group) was conducted at Northwest General Hospital, Peshawar, by reviewing patient records from January 2020 to December 2024. Patients with complete medical records were included consecutively. Group-A was given oral ferrous sulfate (200mg twice daily) for four weeks, whereas Group-B was given intravenous iron sucrose infusion once weekly for the same period. The efficacy was measured in-terms of iron deficiency anemia. The analysis was performed using IBM-SPSS-25. Due to the retrospective design, it inherits the bias of missing data, however exclusion criteria was strictly followed to minimize this bias.

**Results:**

The baseline characteristics, including age, gender, ferritin levels, and CKD stage, were similar across both groups. After four weeks of iron therapy, 24(33.3%) of patients showed improvement in Group-A while 44(61.1%) of patients in Group-B (p=0.001). Sub-Group analyses revealed that Group-B patients show significantly higher improvement in males, older (56-70) years patients, and longer diabetes duration (p<0.001). Stage-5 CKD patients showed better outcomes with IV iron (p<0.001).

**Conclusion:**

IV iron therapy was significantly more effective and show superior efficacy in older patients, males, and those with longer diabetes mellitus duration than oral therapy in improving anemia-related complications in diabetic patients with CKD receiving Erythropoietin, making it a preferable treatment choice in this population.

## INTRODUCTION

Among diabetic patients, chronic kidney disease (CKD) is a serious health issue with studies reporting a high prevalence (20–50%) among type-II diabetics.^1^ In Pakistan, approximately 21.2% of the population is affected by CKD, predominantly males and adults over 50 years old, with diabetic nephropathy as the leading cause.^2^ CKD in diabetic patients often leads to anemia due to decreased Erythropoietin (EPO) production, disrupted iron metabolism, and chronic inflammation. The kidneys’ impaired ability to produce EPO, a hormone critical for red blood cell production, exacerbates cardiovascular risks and diminishes quality of life.^3^ Elevated hepcidin levels in CKD hinder iron absorption, while inflammation disrupts iron utilization and reduces red blood cell lifespan.^3^-^5^ Treatment strategies include recombinant human EPO therapy to stimulate erythropoiesis and intravenous iron supplementation to overcome absorption limitations.^4^,^5^

Iron therapy, either oral or intravenous (IV), is essential for managing anemia associated with CKD. Oral iron, particularly liposomal formulations, has improved bioavailability and tolerance, making it effective in early CKD stages. However, gastrointestinal side effects often lead to reduced adherence.^6^,^7^ Conversely, IV iron therapies, such as iron sucrose and ferric carboxymaltose, provide rapid and sustained iron replenishment without adversely affecting renal function over long-term use.^8^ However, IV iron carries risks, including hypersensitivity reactions, requiring cautious administration.^9^

A study conducted by Khalil and Anees in 2025 compared the efficacy of oral versus intravenous (IV) iron among non-dialysis-dependent CKD patients with iron-deficiency anemia. The study included 88 patients aged 18–70 years, with 44 patients in each group. Group A received oral iron, while Group B received IV iron. Study demonstrate that although both treatments effectively corrected anemia and replenished iron stores, IV iron demonstrated superior efficacy (p<0.0001) in increasing hemoglobin level to 11.88 g/dL ± 0.41 and Ferritin levels to 173.20 ng/mL ± 13.^10^ The efficacy of oral versus IV iron therapy is well established in CKD patients, however its efficacy in diabetic CKD patients is not well explored.

This study addresses the critical challenge of managing iron deficiency anemia in diabetic patients with CKD who are receiving recombinant EPO therapy. While iron therapy is a cornerstone of anemia management, the choice between oral and IV iron remains a key clinical consideration due to differences in efficacy, tolerability, and patient adherence. This study aimed to compare the efficacy of oral versus IV iron therapy in improving hemoglobin levels among this vulnerable population. By identifying the more effective and well-tolerated treatment, the study seeks to provide evidence-based insights to optimize clinical outcomes, enhance patient adherence, and reduce the burden of anemia-associated complications in diabetic CKD patients.

## METHODOLOGY

This retrospective comparative study was carried out at the Department of General Medicine, Northwest General Hospital Peshawar by reviewing five-years patients’ records (January 2020 to December 2024). A non-probability consecutive sampling technique was used to include participants based on predefined inclusion criteria; i.e., patients of either gender, aged between 40 to 70 years, diagnosed with Type-II diabetes, CKD, and iron deficiency anemia while receiving erythropoietin. Patients with decompensated liver cirrhosis, active infections, extensive bleeding requiring blood transfusion, elective surgery during the study period, known hypersensitivity to (oral or IV iron), history of gastrointestinal disorder, hemodialysis dependent, malignancy, pregnant or lactated women were excluded. Furthermore, patients with insufficient medical records or no recorded serum vitamin B12 and folate levels were eliminated to rule out simultaneous B12 or folate deficiencies. Similarly, patients with a known diagnosis of malabsorption syndromes (e.g., coeliac disease, inflammatory bowel disease) or suggestive signs in their clinical history (such as chronic diarrhoea, weight loss, or nutrient deficiency) were excluded to reduce confounding caused by absorption issues.Compliance was determined based on the regular follow-up notes and prescription refill records documented over the 4-week treatment period. Only patients with verified therapy adherence or no mention of non-compliance in physician progress notes were included.

The sample size for our study was calculated using WHO software keeping 95% confidence interval, 80 % power of test, 54.2% effectiveness in IV iron Group-And 31.3% effectiveness in oral iron group.^11^ Expected sample size is 144 patients, 72 patients in each group.

### Ethical approval:

It was obtained from the hospital’s ethical committee (Ref # IRB&EC/2025-GH/2025, dated January 24, 2025).

After approval, patient records have been access from the hospital’s Health Management Information System (HMIS) to identify those who met the inclusion criteria. Only patients with complete medical records were included in the study. Key details such as age, gender, stage of chronic kidney disease, duration of diabetes, and obesity status was noted. Baseline lab results, including complete blood count, liver and kidney function tests, serum electrolytes, and ferritin levels, and chest x-rays were also recorded. Patients were categorized into two groups based on the treatment they had received. Group-A consisted of patients who had been prescribed oral ferrous sulfate (200 mg twice daily) for four weeks, while Group-B included those who had received intravenous iron sucrose infusion (0.5 mg/kg once weekly) for the same duration. Treatment effectiveness was assessed by comparing post-treatment complete blood count results recorded at the end of four weeks.

The patient was labeled diabetic if their fasting blood sugar level exceeding 126 mg/dL, while CKD was characterized by a glomerular filtration rate (GFR) below 60 mL/min/1.73 m². Iron deficiency anemia was identified in patients with a hemoglobin level below 10 g/dL, mean corpuscular volume (MCV) less than 75 fL, and serum ferritin levels under 100 ng/mL. The efficacy of the treatment was assessed in-term of improvement in hemoglobin levels (greater than 11 g/dL) after four weeks of iron therapy, which was considered indicative of effective treatment

Data were recorded using a structured proforma and analyzed with IBM SPSS version 25 for windows (IBM Corp., Armonk, NY, USA). Quantitative variables such as age, baseline hemoglobin levels, serum ferritin levels, and duration of diabetes were summarized using mean ± standard deviations after checking the normality of data through Shapiro Wilk test. Normality was checked to ensure the validity of parametric statistical comparisons through independent sample t test. Categorical variables, including gender, stage of CKD, obesity, and treatment efficacy (defined as hemoglobin levels greater than 11 g/dL after therapy), were summarized as frequencies and percentages. Baseline characteristics was compare by using independent t-test or chi-square test based on nature of the variable (quantitative/qualitative). The effectiveness of the two treatments was compared using the chi-square test, with a p-value ≤0.05 considered statistically significant.

## RESULTS

In our study, a data of total 144 patients were included, with 72 patients in each group. The mean age of participants in Group-A was 56.42 ± 9.20 years, while in Group-B, it was 57.69 ± 9.07 years. Gender distribution revealed a higher proportion of males in both groups. Baseline Hemoglobin levels were 7.81 ± 2.27 ng/mL for Group-A and 8.17 ± 1.19 ng/mL for Group-B (p = 0.371) with a statistically non-significance difference of p=0.371, while Serum ferritin was slightly higher in Group-B. The prevalence of obesity was higher in Group-B. Most of the patients are observed to have CKD Stages-4 and 5 in both groups (Table-I).

**Table-I:** Baseline demographic and clinical characteristics of patients before treatment with oral or intravenous iron.

Demographic and clinical characteristics		Group-A (Oral Iron)	Group-B (IV Iron)	P-Value
Age (years)	(Mean ± SD)	56.42 ± 9.20	57.69 ± 9.07	0.478*
Gender	Male (n,%)	46 (63.9%)	52 (72.2%)	0.284**
	Female (n,%)	26 (36.1%)	20 (27.8%)	
Hemoglobin (g/dL)	(Mean ± SD)	7.81 ± 2.27	8.17 ± 1.19	0.371*
Serum Ferritin (ng/mL)	(Mean ± SD)	68.12 ± 7.24	71.19 ± 6.19	0.41*
Obesity	(n,%)	26 (36.1%)	34 (47.2%)	0.176**
CKD Stage	Stage 3 (n,%)	6 (8.3%)	2 (2.8%)	0.105**
	Stage 4 (n,%)	14 (19.4%)	8 (11.1%)	
	Stage 5 (n,%)	52 (72.2%)	62 (86.1%)	

* p-value is calculated through independent t test,

** p-value is calculated through chi-square test.

After 4-week of treatment plan Group-A showed improvement in 24 (33.3%) patients by achieving hemoglobin level of >11 g/dL , whereas Group-B showed improvement in 44 (61.1%) patients. the difference in both group was statistically significant at p value=0.0001 (Table-II).

**Table-II:** Efficacy of Oral vs Intravenous Iron in Improving Iron Deficiency Anemia in diabetic CKD Patients.

Group	Improved (Hb>11 g/dL)	Not Improved ((Hb<11 g/dL)	p value
Group-A (Oral iron)	24 (33.3%)	48 (66.7%)	0.001
Group-B (Intravenous iron)	44 (61.1%)	28 (38.9%)	

p-value is calculated through chi-square test

Table-III compares the efficacy of oral iron (Group-A) versus IV iron (Group-B) in improving iron deficiency anemia in diabetic CKD patients, with respect to various demographic factors. IV iron showed significant efficacy in improving iron deficiency anemia in diabetic CKD patients compared to oral iron, particularly in older patients (56–70 years, **p < 0.001**), males (**p = 0.001**), those with diabetes for ≥10 years (**p < 0.001**), and patients in CKD Stage-5 (**p < 0.001**). Obese patients also responded better to IV iron (**p = 0.024**).

**Table-III:** Efficacy of oral vs intravenous iron in improving iron deficiency anemia in diabetic ckd patients vs demographic factors.

Category		Group-A (Hb> 11 g/dL)	Group-B (Hb> 11 g/dL)	p-value
Age Groups	40-55 years	12 (35.30%)	10 (41.70%)	0.622
	56-70 years	12 (31.60%)	34 (70.80%)	<0.001
Gender	Male	14 (30.40%)	34 (65.40%)	0.001
	Female	10 (38.50%)	10 (50.00%)	0.439
Duration of Diabetes	<10 years	8 (33.30%)	13 (46.40%)	0.337
	≥10 years	16 (33.30%)	31 (70.50%)	<0.001
Obesity	Yes	10 (38.50%)	23 (67.60%)	0.024
	No	14 (30.40%)	21 (55.30%)	0.022
Stage of CKD	3rd stage	4 (66.70%)	0 (0.00%)	0.104
	4th stage	6 (42.90%)	3 (37.50%)	0.806
	5th stage	14 (26.90%)	41 (66.10%)	<0.001

p-value is calculated through chi-square test.

## DISCUSSION

Our study demonstrated that intravenous iron therapy was significantly effective than oral iron in improving hemoglobin levels and other anemia-related parameters in diabetic patients with CKD receiving erythropoietin. After four weeks of treatment, 61.1% of patients in the IV iron Group-A achieved hemoglobin levels greater than 11 g/dL, compared to only 33.3% in the oral iron group (p=0.001). These findings highlight the superior efficacy of IV iron, likely due to its enhanced bioavailability and rapid replenishment of iron stores, which are particularly beneficial for patients with advanced CKD stages where elevated hepcidin levels impair oral iron absorption.^12^

After four weeks treatment, the hemoglobin level of >11g/dL, was achieved in 33.3% of Group-A (oral iron) patients and 61.1% of Group-B (IV iron) patients. These findings align with existing literature that consistently shows IV iron therapy to be more effective than oral iron in improving anemia-related outcomes. For instance, previous studies report a hemoglobin increase from 6.34 to 11.66 g/dL with IV iron compared to only 6.45 to 9.69 g/dL with oral iron.^13^ Meta-analyses revealed a mean difference of 0.39 g/dL in favor of IV iron. 14 IV therapy also demonstrates superior iron replenishment, with significant increases in ferritin levels (mean difference of 196.81) and transferrin saturation.^14^,^15^ While oral iron is more cost-effective and associated with fewer risks like hypotension, its higher incidence of gastrointestinal side effects 14 and limited efficacy in advanced CKD stages make IV iron a preferable choice for effective anemia management in diabetic patients with CKD receiving erythropoietin.

Studies indicate that female CKD patients, particularly those with uncontrolled diabetes, exhibit a higher prevalence of anemia compared to males, emphasizing the need for gender-specific management strategies.^16^ Poor glycemic control in diabetic patient’s further increases anemia risk, exacerbated by chronic inflammation and reduced erythropoietin production.^17^ Additionally, iron deficiency affects approximately 50% of CKD patients and is associated with increased risks of kidney failure and mortality, significantly impairing quality of life in advanced disease stages.^18^,^19^

A study conducted on older CKD patients revealed that 47% had iron deficiency (ID), which was significantly associated with an increased risk of kidney failure and mortality.^20^ Our research aligns with these findings, demonstrating that IV iron therapy is particularly effective in older patients aged 56–70 years. This subgroup showed marked improvements in hemoglobin levels and anemia-related parameters, likely due to the superior bioavailability and rapid replenishment of iron stores provided by IV therapy. Similarly, males and patients with prolonged diabetes, who often exhibit greater disease severity and chronic inflammation, responded better to IV iron therapy.

Our study also demonstrate that Obese patients benefited significantly from IV therapy, with 67.6% showing improvement compared to 38.5% in the oral iron group (p=0.024). literature suggest the similar findings in obese patients by reporting 67.6% shows improvement with IV iron therapy. The chronic low-grade inflammation associated with obesity may exacerbate functional iron deficiency, making IV therapy a more effective intervention.^21^

Our study emphasizes the stratified analysis of IV iron therapy by CKD stage, demonstrating its significantly greater effectiveness in Stage-5 diabetic CKD patients, with a 66.1% improvement in Group-B compared to 26.9% in Group-A (p<0.001). This stage-specific finding highlights the challenges associated with advanced CKD, such as heightened inflammation, impaired erythropoiesis, and iron homeostasis, which collectively diminish the efficacy of oral iron therapy.^22^ On the other hand, a study by Khalil and Anees 10 provides a broader perspective by showing that serum iron, and serum ferritin levels were significantly higher in the IV iron group than in the oral iron group. Additionally, a significant improvement in haemoglobin levels was observed in the IV iron group across all CKD stages (p<0.0001), while the oral iron group showed no such improvement. Their study concludes that IV iron sucrose therapy is more effective, well-tolerated, and superior to oral iron treatment in addressing iron deficiency anaemia in CKD patients, without a specific focus on individual CKD stages and comorbid.^10^

### Limitations

The study retrospective design, single center nature and limited sample size, prevent it findings generalization to other settings. Also the retrospective design inherit the bias of missing data. However effort was made to minimize this bias by included patients with complete medical record only. The study did not evaluate adherence, cost-effectiveness, or healthcare resource utilization.

## CONCLUSION

Our study confirms that IV iron therapy is significantly effective than oral iron in improving iron deficiency anemia in diabetic patients with CKD receiving erythropoietin. IV iron resulted in higher rates of hemoglobin improvement and better iron replenishment, particularly in patients with advanced CKD, older age, longer diabetes duration, and obesity. These findings underscore the superior bioavailability and efficacy of IV iron, making it a preferred choice for managing iron deficiency anemia in these patients.

### Future recommendation:

Future studies should address these gaps to provide a comprehensive evaluation of therapy impact on diabetic CKD patients by conducting prospective studies with long term follow-up.

### Authors Contribution:

**ND:** Design, supervised, critically revised the manuscript

**AD:** Data collection and Critical Review.

**MKN:** Manuscript writing, responsible and accountable for the accuracy or integrity of the work.

**BH:** Did statistical analysis and wrote Results.

All authors have read and approved the final version.
